# Potential Biomarkers for Disease Stratification and Prognosis Prediction in Pediatric Asthma: LCN2, sST2 and FGF21

**DOI:** 10.1002/kjm2.70137

**Published:** 2025-11-13

**Authors:** Xu Yang, Hai Wang, Yong‐Zheng Zhang, Yan‐Yan Chen, Xin‐Xin Xing, Yi‐Cheng Ding

**Affiliations:** ^1^ Department of Pediatrics II The First Affiliated Hospital of Heilongjiang University of Chinese Medicine Harbin City Heilongjiang Province China; ^2^ Department of Pediatrics The First Affiliated Hospital of Heilongjiang University of Chinese Medicine Harbin City Heilongjiang Province China

**Keywords:** fibroblast growth factor 21, lipocalin‐2, pediatric asthma, prognosis prediction, soluble suppression of tumorigenicity 2

## Abstract

Pediatric asthma is a common chronic airway inflammatory disease, where accurate assessment of disease severity and prognosis prediction is crucial for treatment decisions. Currently, there is a lack of precise and effective biomarkers. This study aimed to investigate the association between serum levels of lipocalin‐2 (LCN2), soluble suppression of tumorigenicity 2 (sST2), and fibroblast growth factor 21 (FGF21) with asthma severity and to evaluate their predictive value for prognosis. A total of 110 asthmatic children (asthma group) were enrolled and stratified by severity into mild (*n* = 50), moderate (*n* = 34), and severe (*n* = 26) subgroups. After 4 weeks of treatment, the asthma group was further divided into good prognosis (*n* = 48) and poor prognosis (*n* = 64) subgroups based on Childhood Asthma Control Test (C‐ACT) scores. Additionally, 110 healthy children were randomly selected as controls. Serum LCN2, sST2, and FGF21 levels were measured using enzyme‐linked immunosorbent assay. Clinical data and pulmonary function parameters were collected. Spearman correlation analysis assessed the relationship between biomarker levels and C‐ACT scores. Multivariate logistic regression identified risk factors for poor prognosis. Receiver operating characteristic (ROC) curve analysis evaluated the predictive performance of LCN2, sST2, and FGF21 for poor prognosis. Serum LCN2, sST2, and FGF21 levels were significantly higher in asthmatic children than in controls (*p* < 0.05), with levels progressively elevating as disease severity increased (*p* < 0.05). The good prognosis group exhibited lower levels of all three biomarkers compared to the poor prognosis group (*p* < 0.05). Spearman analysis revealed negative correlations between biomarker levels and C‐ACT scores (*p* < 0.05). Multivariate logistic regression confirmed LCN2, sST2, and FGF21 as independent risk factors for poor prognosis (*p* < 0.05). ROC analysis demonstrated moderate predictive efficacy for individual biomarkers, while their combination achieved an area under the curve of 0.938, with 93.33% sensitivity and 90.00% specificity, suggesting potential clinical utility for severity assessment and prognosis prediction. Serum LCN2, sST2, and FGF21 levels increase with asthma severity and demonstrate high predictive value for poor prognosis when combined. These biomarkers may serve as early prognostic indicators in pediatric asthma management.

## 1 Introduction

Pediatric bronchial asthma (hereafter referred to as asthma), a prevalent chronic airway inflammatory disease, exhibits high global incidence with a continuing upward trend, posing significant threats to children's physical and mental health as well as quality of life [1, 2]. Pathophysiologically, the disease is characterized by chronic airway inflammation, airway hyperresponsiveness, and reversible airflow limitation [3], and manifests clinically through diverse symptoms including recurrent wheezing, shortness of breath, chest tightness, and coughing [4]. Symptom frequency and severity vary considerably among affected children.

Biomarker identification and application represent a critical research area in pediatric asthma. With the advancing understanding of asthma pathophysiology, increasing efforts focus on discovering novel biomarkers to improve disease stratification and prognostic prediction [5]. Current clinical assessment primarily relies on symptomatic presentation, pulmonary function tests, and conventional laboratory parameters [6, 7], though these methods present limitations [8]. Symptom reporting may be subject to subjective interpretation by children and caregivers, while pulmonary function testing proves challenging in young or severely affected patients, with results potentially confounded by multiple factors. Consequently, identifying more precise and effective biomarkers for pediatric asthma severity classification and prognosis prediction has emerged as a key research priority.

Recent advances in molecular biology have revealed numerous biomarkers associated with disease pathogenesis and progression. Lipocalin‐2 (LCN2) [9], soluble suppression of tumorigenicity 2 (sST2) [10], and fibroblast growth factor 21 (FGF21) [11] have garnered significant attention for their biological functions across various diseases. LCN2, a secretory glycoprotein, modulates inflammatory responses and participates in apoptosis [12]. Studies demonstrate aberrant LCN2 expression in various inflammatory diseases, potentially mediating disease processes through regulation of inflammatory mediators and immune cell activity [13, 14]. Emerging evidence suggests that LCN2 serves as a valuable biomarker for predicting severity and prognosis in respiratory diseases [15, 16, 17]. sST2, the soluble form of the ST2 protein, initially recognized as a cardiovascular biomarker, plays important roles in inflammation and immune regulation [18, 19]. In airway inflammatory diseases such as asthma, sST2 expression levels may change and contribute to the regulation of airway inflammation [20]. FGF21, primarily secreted by the liver, regulates glucose/lipid metabolism and insulin sensitivity [21]. Recent studies indicate its involvement in inflammatory and metabolic disorders through modulation of metabolic status and immune responses [22, 23], with growing interest in its association with pulmonary inflammation and injury [24, 25].

Despite the established biological roles of LCN2, sST2, and FGF21 in various diseases, their potential in pediatric asthma stratification and prognosis prediction remains underexplored. This study investigates the association between serum levels of these biomarkers and asthma severity while evaluating their prognostic value. Elucidating the mechanistic roles of these biomarkers may provide novel approaches for clinical assessment and prognostic prediction in pediatric asthma, ultimately supporting evidence‐based therapeutic decisions to improve patient outcomes and quality of life.

## 2 Materials and Methods

### 2.1 Study Subjects

A total of 110 children with acute asthma exacerbations admitted to our hospital between December 2022 and June 2024 were enrolled. Inclusion criteria for the asthma group: (1) Patients aged 6–14 years; (2) Patients diagnosed with asthma according to the 2016 Guidelines for the Diagnosis and Optimal Management of Childhood Asthma [26]; (3) Patients without other respiratory diseases; (4) Patients and guardians capable of cooperating with required examinations; (5) Patients with complete clinical data. Exclusion criteria for the asthma group: (1) Patients with wheezing caused by viral, bacterial, or mycoplasma infections; (2) Patients with severe cardiac, hepatic, renal, or hematological diseases; (3) Patients who had received antiasthmatic drugs, antiallergic agents, immunosuppressants, immunomodulators, or inflammatory mediator antagonists alone or in any combination within the past month; (4) Patients with congenital heart disease, bronchial foreign bodies, tuberculosis infection, or respiratory failure; (5) Patients with infectious diseases; (6) Patients with cognitive impairment unable to cooperate with study procedures. An additional 110 healthy children undergoing routine physical examinations during the same period served as controls. Inclusion criteria for the control group: (1) Patients with normal physical examinations; (2) Patients with normal serum biochemical parameters. The control group exclusion criteria matched those of the asthma group. Written informed consent was obtained from all participants' parents or legal guardians. The present study was approved by the Ethics Committee of The First Affiliated Hospital of Heilongjiang University of Chinese Medicine (No. 202011HB36) and written informed consent was provided by all patients prior to the study start. All procedures were performed in accordance with the ethical standards of the Institutional Review Board and The Declaration of Helsinki, and its later amendments or comparable ethical standards.

## 3 Methods

### 3.1 Clinical Data Collection

Clinical data were collected for all enrolled patients, including demographic and clinical characteristics such as age, gender, BMI, family history, and history of rhinitis. Patients were stratified into mild (*n* = 50), moderate (*n* = 34), and severe (*n* = 26) groups based on disease severity using standardized criteria: Mild asthma: Symptoms occurring ≥ 1 time/week with possible impact on activity and sleep; nocturnal symptoms > 2 times/month; Forced expiratory volume in 1 s (FEV_1_) ≥ 70% predicted; and peak expiratory flow (PEF) or FEV_1_ variability of 20%–30%. Moderate asthma: Daily symptoms with significant impact on activity and sleep; nocturnal symptoms ≥ 2 times/week; FEV_1_ 60%–79% predicted or meeting the criterion of 60% ≤ FEV_1_% predicted ≤ 69%. Severe asthma: Persistent daily symptoms; frequent nocturnal symptoms; substantial limitation of physical activity; PEF < 60% predicted; PEF or FEV_1_ variability > 30%; and FEV_1_ 35%–49% predicted.

### 3.2 Pulmonary Function Testing

Pulmonary function parameters were measured in asthmatic children on admission using a spirometer (COSMED, Italy; model: Quark PFT4). Following bronchodilator inhalation, subjects performed maximal forced expiratory maneuvers to determine peak expiratory flow percentage of predicted (PEF% pred) and forced expiratory volume in 1 s percentage of predicted (FEV_1_% pred). Prior to testing, all participants received standardized breathing technique instructions, with each child completing ≥ 3 reproducible maneuvers.

### 3.3 Blood Sample Collection and Analysis

All participants fasted overnight (from 22:00), with venous blood collected between 09:00 and 10:00 the following morning (2 mL per tube). One tube was maintained at room temperature for 30 min, followed by centrifugation at 4°C (3000 rpm for 15 min) to isolate serum, which was stored at −20°C. Serum Immunoglobulin E (IgE) and CRP levels were quantified using an automated protein analyzer (Siemens AG, Germany), while eosinophil counts were determined using a Sysmex XN9000 automated hematology analyzer (Sysmex Corporation, Japan). The second blood tube was processed identically, with serum stored at −80°C for subsequent analysis. Serum concentrations of LCN2, sST2, and FGF21 were measured using commercial enzyme‐linked immunosorbent assay kits (Elabscience Biotechnology Co., China) via sandwich immunoassays, with all procedures performed strictly according to manufacturer protocols.

### 3.4 Prognostic Evaluation of Asthma Control Status

Asthmatic children received standardized treatment for 4 weeks, followed by assessment using the Childhood Asthma Control Test (C‐ACT) [27]. The C‐ACT comprises seven items with a total score ranging from 0 to 27: Scores ≥ 20 indicated well‐controlled asthma; scores of 15–19 denoted suboptimal control (requiring therapeutic reevaluation); and scores < 15 signified poor control (necessitating immediate treatment adjustment). Based on C‐ACT scores, patients were stratified into two groups: the poor prognosis group (C‐ACT < 20, *n* = 26) and the good prognosis group (C‐ACT ≥ 20, *n* = 70).

### 3.5 Statistical Analysis

Data analysis was performed using Statistical Package for the Social Sciences 24.0 (IBM Corp., USA). The normality of continuous variables was assessed by the Shapiro–Wilk test, with normally distributed data presented as mean ± SD and compared using independent *t* tests. Categorical variables were analyzed by *χ* ^2^ or corrected *χ* ^2^ tests. Spearman's correlation coefficient evaluated bivariate associations, while independent predictors were identified through multivariate logistic regression. Receiver operating characteristic (ROC) curve analysis determined the area under the curve (AUC) and optimal cutoff values (based on the Youden index). All tests employed a two‐sided significance level of *α* = 0.05.

## 4 Results

### 4.1 Demographic Characteristics Analysis

Analysis of baseline characteristics among asthma severity groups and healthy controls revealed no significant differences in age, gender, BMI, or family history of asthma among mild, moderate, and severe asthma groups (*p* = 0.173, 0.679, 0.643, and 0.707, respectively), suggesting the limited value of these parameters for severity stratification. In contrast, significant differences were observed in C‐ACT scores, pulmonary function parameters (PEF% pred and FEV_1_% pred), and laboratory markers (C‐reactive protein, IgE, and eosinophil counts) between asthma severity groups and healthy controls (all *p* < 0.001, 0.007, or 0.002). A progressive deterioration pattern was noted from mild to severe asthma, characterized by decreasing C‐ACT scores, declining PEF% pred and FEV_1_% pred values, and elevated levels of C‐reactive protein, IgE, and eosinophils. These findings demonstrate significant associations between these parameters and asthma severity, highlighting their clinical relevance for pediatric asthma assessment. The results provide a foundation for evaluating LCN2, sST2, and FGF21 as potential biomarkers to enable comprehensive disease characterization (Table 1).

**TABLE 1:** Demographic and clinical characteristics of asthmatic children stratified by disease severity.

Parameter	Mild group (*n* = 50)	Moderate group (*n* = 34)	Severe group (*n* = 26)	Healthy controls (*n* = 110)	*p* (ANOVA among severity groups)
Age (years)	8 (8, 10)	9 (8, 10)	8 (7, 9)	8 (7, 10)	0.173
Gender					0.679
Male	31 (62.00%)	18 (52.94%)	16 (61.54%)	54 (56.25%)	
Female	19 (38.00%)	16 (47.06%)	10 (38.46%)	42 (43.75%)	
BMI	18.22 ± 2.32	18.46 ± 2.06	18.72 ± 2.24	18.5 ± 3.21	0.643
Family history of asthma	8 (16.00%)	7 (20.59%)	6 (23.08%)	13 (13.54%)	0.707
C‐ACT score	24.5 (22, 26)	20 (18, 22)	16 (15, 18)	/	< 0.001
PEF% predicted	81.95 ± 9.49	77.63 ± 11.24	74.23 ± 10.36	92.56 ± 6.28	0.007
FEV_1_% predicted	88.54 ± 8.56	85.74 ± 9.41	80.32 ± 10.23	92.55 ± 5.32	0.002
Laboratory parameters					
CRP (mg/L)	10.84 ± 2.12	14.53 ± 2.71	16.44 ± 2.25	6.79 ± 1.56	< 0.001
IgE (IU/mL)	165.02 ± 23.56	185.74 ± 26.74	201.45 ± 28.77	59.13 ± 14.52	< 0.001
EOS (10^9^/L)	0.66 ± 0.12	0.74 ± 0.14	0.89 ± 0.15	0.520 ± 0.13	< 0.001

Abbreviations: ANOVA = analysis of variance; BMI = body mass index; CRP = C‐reactive protein; EOS = eosinophils; FEV_1_% pred = forced expiratory volume in 1 s percentage of predicted value; IgE = immunoglobulin E; PEF% pred = peak expiratory flow percentage of predicted value.

### 4.2 Serum Levels of LCN2, sST2, and FGF21 in Asthma and Control Groups

**FIGURE 1:**
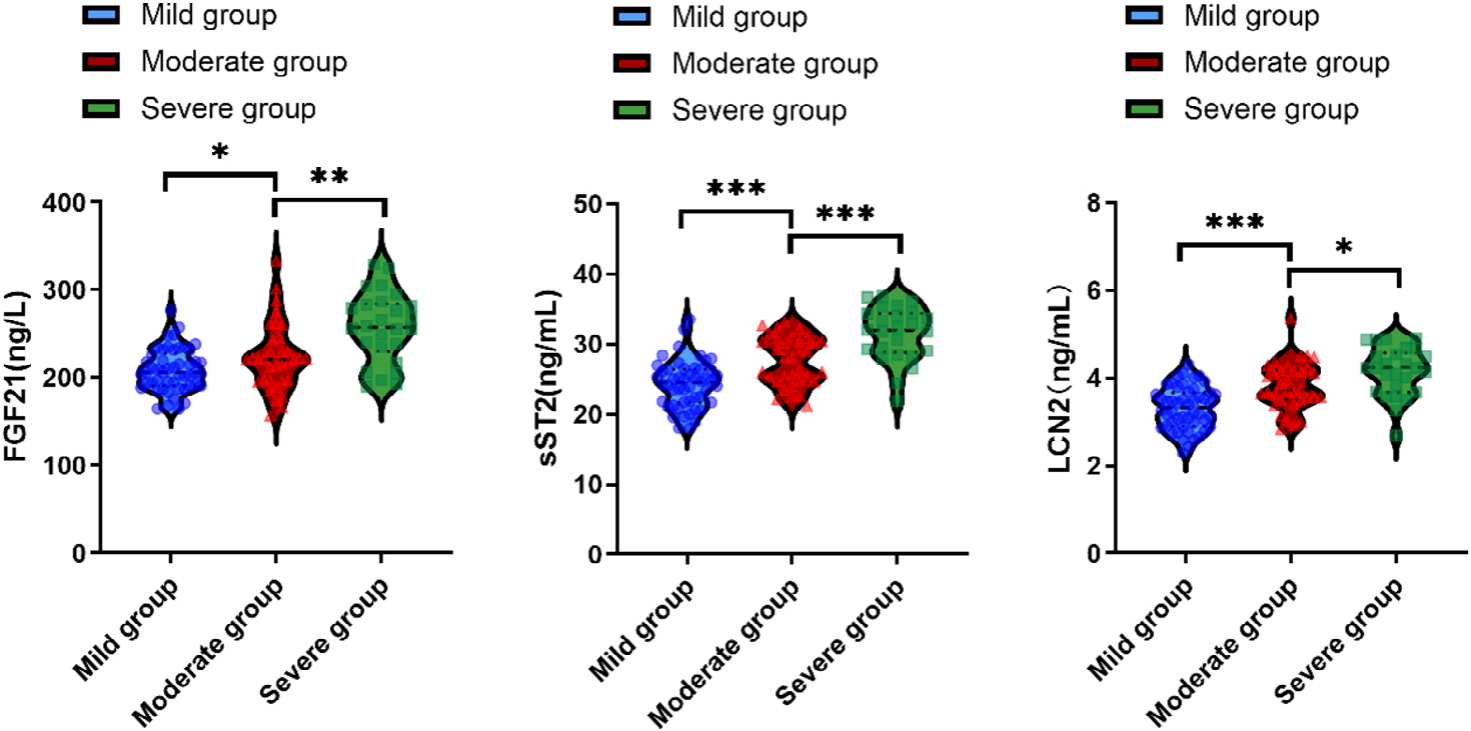
Serum LCN2, sST2, and FGF21 expression levels in asthmatic children with different disease severity grades. *p < 0.05,**p < 0.01, ***p < 0.001.

**FIGURE 2:**
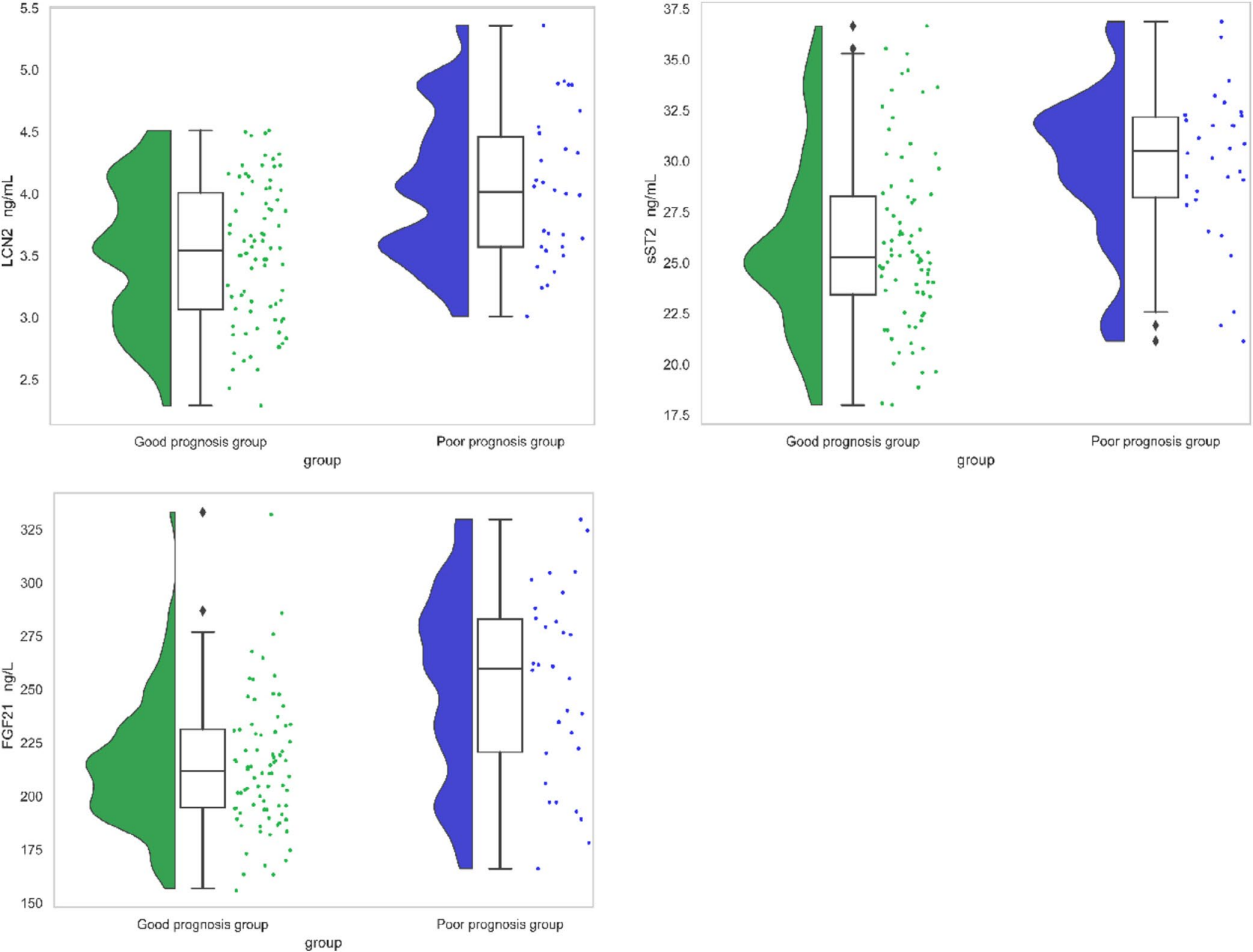
Serum LCN2, sST2, and FGF21 expression levels in asthmatic children with favorable and unfavorable prognosis.

The study population comprised four groups: mild asthma (*n* = 50), moderate asthma (*n* = 34), severe asthma (*n* = 26), and healthy control (*n* = 110) groups. Serum LCN2 levels were 3.34 ± 0.52 ng/mL (mild), 3.85 ± 0.67 ng/mL (moderate), 4.23 ± 0.60 ng/mL (severe), and 2.86 ± 0.46 ng/mL (controls), demonstrating progressive elevation with increasing asthma severity (all *p* < 0.05 versus controls). sST2 concentrations showed similar trends: 23.54 ± 3.55 ng/mL (mild), 27.48 ± 3.84 ng/mL (moderate), 31.75 ± 3.73 ng/mL (severe), versus 14.76 ± 3.47 ng/mL (controls) (all *p* < 0.05). FGF21 levels exhibited comparable patterns: 184.63 ± 26.54 ng/L (mild), 234.72 ± 31.78 ng/L (moderate), 287.33 ± 39.45 ng/L (severe), versus 57.46 ± 16.74 ng/L (controls) (all *p* < 0.05). These results suggest potential associations between biomarker levels and disease severity (Table 2 and Figure 1). When stratified by prognosis (good prognosis *n* = 80; poor prognosis *n* = 30), significant differences emerged: LCN2 levels were higher in poor prognosis (4.03 ± 0.60 ng/mL) than in the good prognosis (3.51 ± 0.57 ng/mL) groups (*p* < 0.001). sST2 concentrations differed significantly between poor prognosis (median: 30.51 ng/mL, IQR: 28.10–32.24) and good prognosis (median: 25.27 ng/mL, IQR: 23.41–28.30) groups (*p* < 0.001). Similarly, FGF21 levels were elevated in poor prognosis (median: 259.92 ng/L, IQR: 220.17–283.27) compared to the good prognosis (median: 211.80 ng/L, IQR: 194.69–231.51) groups (*p* < 0.001). These results indicate potential prognostic value for all three biomarkers (Table 3 and Figure 2).

**TABLE 2:** Comparison of serum LCN2, sST2, and FGF21 levels among asthmatic children stratified by disease severity.

Biomarker	Mild group (*n* = 50)	Moderate group (*n* = 34)	Severe group (*n* = 26)	Healthy controls (*n* = 110)	*p* (mild vs. moderate)	*p* (moderate vs. severe)
LCN2	3.34 ± 0.52	3.85 ± 0.67	4.23 ± 0.60	2.86 ± 0.46	< 0.001	0.026
sST2	23.54 ± 3.55	27.48 ± 3.84	31.75 ± 3.73	14.76 ± 3.47	< 0.001	< 0.001
FGF21	184.63 ± 26.54	234.72 ± 31.78	287.33 ± 39.45	57.46 ± 16.74	0.029	0.002

**TABLE 3:** Comparison of serum LCN2, sST2, and FGF21 levels between asthmatic children with favorable vs. unfavorable prognosis.

Biomarker	Good prognosis group (*n* = 80)	Poor prognosis group (*n* = 30)	*p*
LCN2 (ng/mL)	3.51 ± 0.57	4.03 ± 0.599	< 0.001
sST2 (ng/mL)	25.27 (23.41, 28.30)	30.51 (28.1, 32.24)	< 0.001
FGF21 (ng/L)	211.80 (194.69, 231.51)	259.92 (220.17, 283.27)	< 0.001

### 4.3 Correlation Between Serum LCN2, sST2, FGF21 Levels and C‐ACT Scores in Asthmatic Children

**FIGURE 3:**
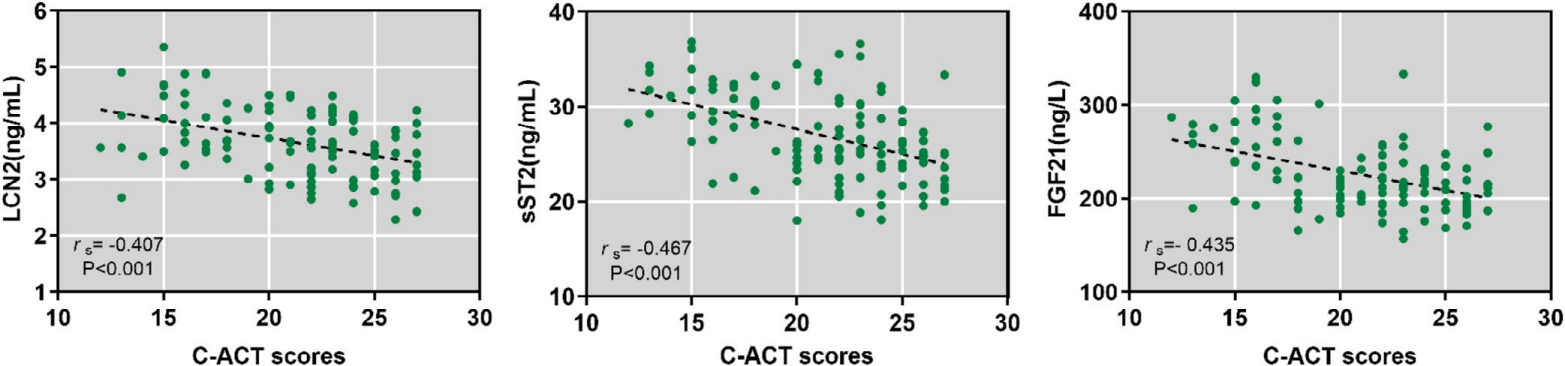
Correlation analysis between serum LCN2, sST2, FGF21 levels and C‐ACT scores in asthmatic children.

Spearman correlation analysis revealed significant negative correlations between serum levels of LCN2, sST2, FGF21, and C‐ACT scores (*r*_s_ = −0.407, −0.467, and −0.435, respectively; all *p* < 0.001) (Figure 3).

### 4.4 Multivariate Logistic Regression Analysis of Poor Prognosis in Asthmatic Children

Using poor prognosis (no = 0, yes = 1) as the dependent variable (with adjustment for gender, age, and BMI), multivariate logistic regression demonstrated that elevated serum levels of LCN2 (OR = 2.862, 95% confidence interval [95% CI]: 1.100–7.442), sST2 (OR = 1.227, 95% CI: 1.071–1.406), and FGF21 (OR = 1.023 95% CI: 1.009–1.038) were independent risk factors for poor prognosis in childhood asthma (all *p* < 0.05) (Table 4).

**TABLE 4:** Multivariate logistic regression analysis of poor prognosis in asthmatic children.

Variable	*β*	SE	Wald *χ* ^2^	*p*	OR (95% CI)
Constant	−17.104	4.547	14.1536	< 0.001	—
Age	0.040	0.178	0.051	0.821	1.041 (0.743–1.476)
Gender	−1.083	0.588	3.397	0.065	0.339 (0.107–1.071)
BMI	0.073	0.139	0.277	0.598	1.076 (0.819–1.414)
LCN2	1.051	0.488	4.649	0.031	2.862 (1.100–7.442)
sST2	0.205	0.069	8.704	0.003	1.227 (1.071–1.406)
FGF21	0.023	0.007	9.694	0.002	1.023 (1.009–1.038)

### 4.5 Predictive Performance of Serum LCN2, sST2, and FGF21 for Poor Prognosis in Asthmatic Children

**FIGURE 4:**
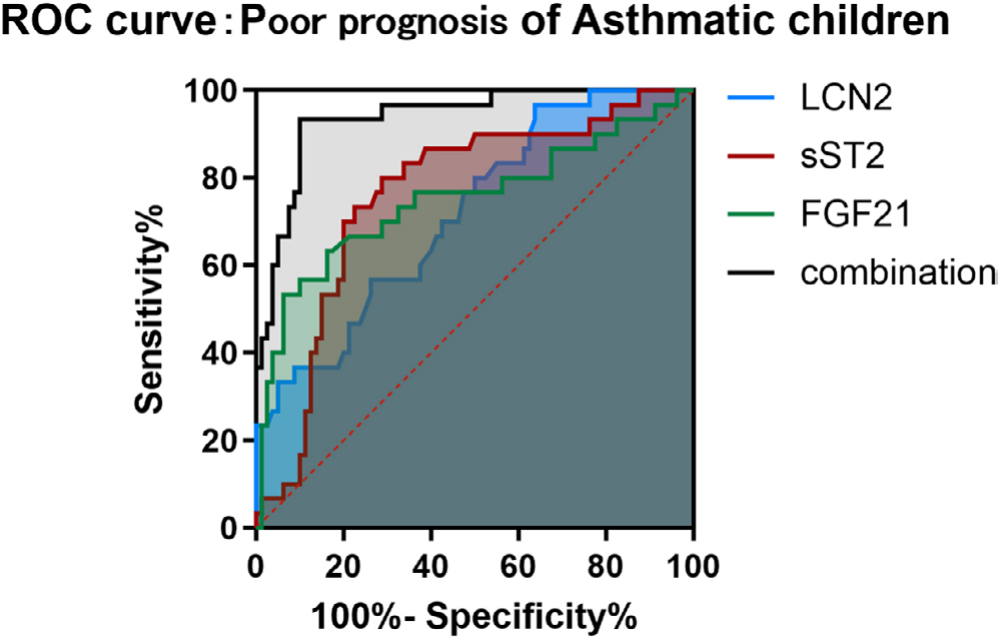
ROC curves for predicting poor prognosis in asthmatic children by serum LCN2, sST2, FGF21, and their combination.

ROC curve analysis evaluating the predictive value of serum biomarkers for poor asthma prognosis showed: LCN2: AUC = 0.719 (95% CI: 0.615–0.822, *p* < 0.001), optimal cutoff = 3.23 ng/mL (sensitivity = 96.67%, specificity = 36.25%); sST2: AUC = 0.762 (95% CI: 0.663–0.862, *p* < 0.001), optimal cutoff = 27.75 ng/mL (sensitivity = 80.00%, specificity = 71.25%); FGF21: AUC = 0.751 (95% CI: 0.634–0.868, *p* < 0.001), optimal cutoff = 238.50 ng/L (sensitivity = 63.33%, specificity = 83.75%). The combined biomarker model exhibited superior predictive performance (AUC = 0.938, 95% CI: 0.890–0.985; *p* < 0.001, sensitivity = 93.33%, specificity = 90.00%) (Table 5 and Figure 4).

**TABLE 5:** Predictive performance of serum LCN2, sST2, FGF21, and their combination for poor prognosis in asthmatic children.

Biomarker	AUC (95% CI)	*p*	Optimal cut‐off	Sensitivity	Specificity
LCN2	0.719 (0.615–0.822)	< 0.001	3.23 ng/mL	96.67%	36.25%
sST2	0.762 (0.663–0.862)	< 0.001	27.75 ng/mL	80.00%	71.25%
FGF21	0.751 (0.634–0.868)	< 0.001	238.50 ng/L	63.33%	83.75%
Combined	0.938 (0.890–0.985)	< 0.001	/	93.33%	90.00%

## 5 Discussion

Bronchial asthma is the most common chronic airway inflammatory disease in childhood, with its global incidence showing an increasing trend, particularly in developing countries [28]. The heterogeneous nature of asthma's pathological features and complex pathogenesis pose significant challenges for early clinical diagnosis, severity stratification, and prognostic evaluation [29]. Previous studies have established the clinical utility of peripheral blood biomarkers for asthma phenotyping, disease severity assessment, and outcome prediction [5]. This study investigates serum levels of LCN2, sST2, and FGF21 in pediatric asthma patients to evaluate their clinical correlations with disease severity and prognosis. Through systematic analysis of their associations with disease classification and prognosis, this study provides multidimensional evidence supporting their potential clinical application as biomarkers.

Inflammation and airway remodeling remain central features in asthma pathogenesis research [30, 31, 32]. Consistent with previous biomarker studies, serum levels of LCN2, sST2, and FGF21 were significantly elevated in asthmatic children compared to healthy controls, aligning with findings of dysregulated inflammatory proteins in asthma patients. For instance, multiple cytokines and inflammatory mediators participate in airway inflammation. LCN2, an inflammatory protein, is likely secreted by airway epithelial cells and immune cells under inflammatory stimulation, thereby amplifying inflammatory cascades and exacerbating airway pathology [33]. Suppression of tumorigenicity 2 (ST2) has been proposed as a receptor contributing to neutrophilic inflammation in patients with type 2‐low asthma [34]. Increased serum levels of sST2, a soluble IL‐33 receptor, lead to enhanced neutrophilic lung disease [35], which aligns with the elevated sST2 levels observed in asthmatic children in this study. Emerging evidence suggests that FGF receptor inhibition attenuates neutrophilic inflammation in steroid‐resistant severe asthma models, highlighting FGF as a potential therapeutic target [34]. Liu et al. demonstrated increased FGF21 levels in ovalbumin‐induced asthmatic mice [35]. The elevated FGF21 levels observed in pediatric asthma patients may reflect metabolic dysregulation and systemic stress responses associated with asthma. Collectively, these findings underscore the pathogenic significance of these three biomarkers in asthma development and progression.

Results demonstrated significantly elevated serum levels of LCN2, sST2, and FGF21 in asthmatic children compared to healthy controls, with levels increasing progressively with disease severity. Wang et al. reported increased plasma LCN2 levels in asthma patients, showing a negative correlation with emphysema extent—suggesting LCN2 as a potential biomarker for differentiating Asthma‐COPD Overlap Syndrome from asthma alone [16]. Another study observed higher serum LCN2 in asthmatic children, with the most severe cases exhibiting the highest levels, supporting its role as a severity marker [36]. Elevated serum sST2 may promote Th2 inflammation, exacerbating asthma and food allergy severity [37]. In asthmatic lungs, IL‐33 is expressed by airway smooth muscle cells, serving as both an inflammatory marker and therapeutic target. As a decoy receptor, sST2 binds IL‐33, inhibiting IL‐33‐induced nuclear factor kappa‐light‐chain‐enhancer of activated B cells activation and downstream cytokines (IL‐4, IL‐5, IL‐13) [38, 39]. Recent evidence indicates serum FGF21 levels negatively correlate with lung function in asthma patients, implicating metabolic dysregulation in airway inflammation [40]. Prognostic analysis revealed significantly higher serum LCN2, sST2, and FGF21 levels in children with poor prognosis (based on C‐ACT scores) versus those with favorable outcomes. Spearman correlation confirmed negative associations between all three biomarkers and C‐ACT scores, linking their elevation to poor asthma control (correlation coefficients: −0.407, −0.467, and −0.435, respectively; all *p* < 0.001). Multivariate logistic regression identified each biomarker as an independent risk factor for poor prognosis (OR > 1). ROC curve analysis showed that the combined detection of the three biomarkers achieved an AUC of 0.938, with a sensitivity of 93.33% and specificity of 90.00%, significantly outperforming single‐marker assessments. These results directly support the superior predictive accuracy of a multi‐marker strategy. The combined detection of LCN2, sST2, and FGF21 may improve prognostic accuracy by comprehensively reflecting the inflammatory, metabolic, and repair states of asthma.

However, this study has limitations compared to large‐scale, multicenter, high‐quality investigations. The sample originated from a single center and was relatively small, potentially limiting generalizability due to the lack of geographic, ethnic, and environmental diversity, contrasting with the representative advantage of multicenter samples. The follow‐up period of only 4 weeks post‐treatment was shorter than long‐term studies, hindering a comprehensive assessment of long‐term outcomes and the relationship between biomarkers and disease progression. Furthermore, the study focuses on associations between biomarkers and disease severity/prognosis without exploring the underlying mechanisms in depth, representing a mechanistic limitation compared to research investigating asthma pathogenesis from a molecular biology perspective. Future studies should adopt robust research models, conducting multicenter investigations with larger samples, extending follow‐up duration, and integrating basic experiments to elucidate the mechanistic roles of these three biomarkers in asthma pathogenesis, thereby strengthening the theoretical and practical basis for precision diagnosis and treatment of pediatric asthma.

In summary, this study confirms that serum levels of LCN2, sST2, and FGF21 are significantly associated with disease severity and prognosis in childhood asthma, and their combined detection substantially improves prognostic accuracy. By elucidating the clinical relevance of LCN2, sST2, and FGF21, this work not only provides new tools for disease assessment and outcome prediction in pediatric asthma, but also establishes a foundation for personalized treatment, phenotype classification, and targeted intervention. Although further validation through multicenter studies is needed, the current data strongly support the clinical application of these biomarkers, offering the potential to advance asthma management from empirical treatment toward precision medicine.

## Ethics Statement

The present study was approved by the Ethics Committee of The First Affiliated Hospital of Heilongjiang University of Chinese Medicine (No. 202011HB36) and written informed consent was provided by all patients prior to the study start. All procedures were performed in accordance with the ethical standards of the Institutional Review Board and The Declaration of Helsinki, and its later amendments or comparable ethical standards.

## Conflicts of Interest

The authors declare no conflicts of interest.

## Data Availability

Data is available from the corresponding author on request.
